# Retrospective analysis of COVID-19 patients with Guillain–Barre, Miller–Fisher, and opsoclonus–myoclonus–ataxia syndromes—a case series

**DOI:** 10.1007/s10354-023-01018-4

**Published:** 2023-07-31

**Authors:** Elisabeth Olbert, Naela Alhani, Walter Struhal

**Affiliations:** 1 Karl Landsteiner University of Health Sciences, Dr. Karl-Dorrek-Straße 30, 3500 Krems, Austria; 2 Department of Neurology, University Hospital Tulln, Alter Ziegelweg 10, 3430 Tulln an der Donau, Austria

**Keywords:** COVID, Post-COVID syndrome, Guillain–Barre syndrome, Opsoclonus–myoclonus syndrome, Miller–Fisher syndrome

## Abstract

**Background:**

In accordance with the rising number of SARS-CoV‑2 infections, reports of neurological complications have also increased. They include cerebrovascular diseases but also immunological diseases such as Guillain–Barre syndrome (GBS), Miller–Fisher syndrome (MFS), and opsoclonus–myoclonus–ataxia syndrome (OMAS). While GBS and MFS are typical postinfectious complications, OMAS has only recently been described in the context of COVID-19. GBS, MFS, and OMAS can occur as para- and postinfectious, with different underlying pathomechanisms depending on the time of neurological symptom onset. The study aimed to describe clinical features, time between infection and onset of neurological symptoms, and outcome for these diseases.

**Methods:**

All COVID-19 patients treated in the neurological ward between January 2020 and December 2022 were screened for GBS, MFS, and OMAS. The clinical features of all patients, with a particular focus on the time of onset of neurological symptoms, were analyzed.

**Results:**

This case series included 12 patients (7 GBS, 2 MFS, 3 OMAS). All GBS and one MFS patient received immunomodulatory treatment. Three patients (2 GBS, 1 OMAS) had a severe COVID-19 infection and received mechanical ventilation. In patients with OMAS, only one patient received treatment with intravenous immunoglobulin and cortisone. The remaining two patients, both with disease onset concurrent with SARS-COV‑2 infection, recovered swiftly without treatment. In all subgroups, patients with concurrent onset of neurological symptoms and COVID-19 infection showed a trend toward shorter disease duration.

**Conclusion:**

All patient groups displayed a shorter disease duration if the onset of neurological symptoms occurred shortly after the COVID-19 diagnosis. In particular, both the OMAS patients with symptom onset concurrent with COVID-19 showed only abortive symptoms followed by a swift recovery. This observation would suggest different pathomechanisms for immune-mediated diseases depending on the time of onset after an infection.

**Supplementary Information:**

The online version of this article (10.1007/s10354-023-01018-4) contains supplementary material, which is available to authorized users.

## Background

During the COVID-19 pandemic, neurological symptoms and diseases associated with a severe acute respiratory syndrome coronavirus 2 (SARS-CoV-2) infection were reported in the literature and repeatedly observed in clinical work. These include smell and taste disorders, cerebrovascular diseases, encephalopathy, and demyelinating diseases [1]. In addition, rare immune-mediated diseases such as Guillain–Barre syndrome (GBS), Miller–Fisher syndrome (MFS), and opsoclonus–myoclonus–ataxia syndrome (OMAS) were reported [2–5].

GBS is a postinfectious, immune-mediated, monophasic, and demyelinating neuropathy and was repeatedly observed in the context of an acute or recent SARS-CoV‑2 infection. It seems that GBS is more frequent in patients with an acute or recent SARS-CoV‑2 infection than in noninfected patients, although the data on this are insufficient. For example, the incidence of GBS in COVID-19 patients increased compared to non-COVID controls in the first wave of the pandemic in Italy. This effect could be explained by a higher risk of GBS in COVID-19 patients and a lower incidence of other infectious diseases during that time [6]. The incidence rate of GBS in COVID patients is estimated to be between 0.5 and 0.05 per 1000 COVID-19 infections, depending on the calculation method applied [6]. This estimation is higher than, for example, the incidence of GBS after *Campylobacter jejuni* infection (0.25–0.65 per 1000 cases), a typical trigger for GBS [6].

Typically, GBS is caused by a postinfectious mechanism mediated through molecular mimicry, and the presence of antiganglioside antibodies is frequent [7]. In non-COVID-19 patients, GBS has a typical onset 1–3 weeks after an infectious disease [8, 9]. In COVID-19-associated GBS, however, a para- and postinfectious onset has been described. Nevertheless, a postinfectious onset in COVID-19-associated GBS is more common than a parainfectious onset [8]. However, the differentiation is not easy to achieve in the context of SARS-CoV2 as the onset of COVID-19 is often unknown and the incubation period may be up to 14 days [8, 9]. Due to these differences in the onset of GBS, other pathomechanisms have been discussed for parainfectious COVID-19-associated GBS cases. This is because a sufficient time between infection and antibody generation, leading to molecular mimicry, is needed for the development of postinfectious GBS. Other possible pathomechanisms for parainfectious GBS include direct damage through the virus or dysregulated inflammatory response, possibly associated with increased cytokines and cell-mediated immunity. In postinfectious COVID-19-associated GBS, an antibody-mediated mechanism seems common, and anti-GM1 IgG antibodies can be found in these patients. This is supported by the finding that auto-antibodies seem less likely in COVID-19-associated GBS than in non-COVID cases and could explain the shorter interval between infection and GBS onset [1, 7, 10].

MFS is a variant of GBS and is defined as a monophasic, demyelinating cranial neuropathy [10]. The current evidence of COVID-19-associated MFS is based on case reports. The mean time for the onset of MFS symptoms was around 15 days after COVID-19 diagnosis. Individual case reports describe an onset concurrent with COVID-19 diagnosis [11]. Intravenous immunoglobulin (IVIG) therapy was typically used, and the patients made a full recovery within 2 weeks [12, 13]. Anti-GQ1b IgG antibodies are present in most MFS patients, while a lower frequency of antibodies is seen in COVID-19-associated MFS. Similar to the aforementioned pathomechanism in GBS, this could suggest against molecular mimicry as the main driver of the disease in MFS. This is supported by a shorter duration between MFS symptom onset after COVID-19 infection in some patients and could suggest cell-mediated immunity as an underlying mechanism [7].

Another complication after COVID-19 infection is OMAS, which was primarily known as a paraneoplastic syndrome in the past but can occur after different infections, including herpes viruses, arbovirus, or parasitic infections [5, 14, 15]. Several case reports have shed light on COVID-19-associated OMAS cases [4]. Symptom onset ranges from 7 to 12 days after COVID-19 onset, and symptom severity varies from complete OMAS to abortive syndrome without myoclonus [5]. In addition, some patients reported encephalopathy or cognitive impairment and other neurological deficits [5, 16]. Depending on the literature, therapy varies between cortisone, plasmapheresis, rituximab, and IVIG, with cortisone being the most widely used substance. All patients made a swift recovery within weeks [17–21]. One case with associated anti-GFAP antibodies was reported [22]. Although the pathophysiology behind COVID-19-associated OMAS is still unknown, an immune-mediated mechanism is suggested due to the rapid improvement after immunotherapy, such as steroids, IVIG, or rituximab [5].

Concluding, all three diseases can be para- and post-infectious with presumed different mechanisms depending on the time of onset after the infection.

The aim of this study was to describe clinical features of patients with GBS, MFS, and OMAS with concurrent or previous COVID-19 disease placing special focus on the time of onset of neurological symptoms after SARS-CoV‑2 infection.

## Methods

Patients treated as inpatients in the neurological ward of the University Hospital Tulln from January 2020 to December 2022, diagnosed with either an acute or recent SARS-CoV‑2 infection, were retrospectively screened for this analysis. All patients diagnosed with GBS, MFS, and OMAS were included in this case series. No patient was excluded from the study.

All patients underwent a neurological work-up, depending on their individual clinical presentation, including magnetic resonance imaging (MRI) or computed tomography (CT) scan of the brain as well as lumbar puncture. Patients with OMAS underwent tumor screening as well. Antibody (AB) testing included ganglioside antibodies (GM1-, GQ1b‑, GD1a‑, GD1b-AB) for GBS and MFS patients and onconeuronal and paraneoplastic antibodies (Hu‑, Yo‑, Ri‑, Amphiphysin‑, Ma1/2-, GAD65-, NMDAR-, AMPAR-, GABAR-, Glycine receptor-AB) in patients with OMAS.

Treatment varied between cases depending on symptoms, severity, and comorbidities. The clinical severity of COVID-19 in each patient was graded as mild, moderate, and severe using the disease severity classification. Mild disease is defined as a symptomatic disease course without evidence of pneumonia or hypoxia, moderate disease with evidence of pneumonia, and severe disease is defined as pneumonia with signs of hypoxia or severe respiratory distress [23]. The study was approved by the local ethics committee (Commission for Scientific Integrity and Ethics, Karl Landsteiner University of Health Sciences, EK Nr: 1076/2022).

## Results

Overall, 105 patients were screened for this study, and 12 patients were included in the analysis (Supplementary table). These included seven patients diagnosed with GBS, two patients with MFS, one with OMAS, and two with incomplete OMAS symptoms. During the same period, ten non-COVID-associated patients with GBS were treated at the hospital.

In GBS patients, symptom onset varied from concurrent with COVID-19 onset until 2 weeks after COVID-19 infection. All patients exhibited increased protein in cerebrospinal fluid (CSF); only one had positive GT1A antibodies. Three patients were treated with IVIGs and one with cortisone due to suspected chronic inflammatory demyelinating polyneuropathy. All patients experienced a significant improvement in symptoms over the course of 6 months.

In the MFS group, both patients experienced onset together with COVID-19 infection. One patient was treated with IVIG and fully recovered within 1 week. The other patient did not require immunomodulatory treatment due to a swift recovery but presented with mild residual symptoms after 1 month and positive anti-GT1A IgG and anti-GQ1b antibodies.

In patients with OMAS, clinical presentations varied considerably. One patient showed signs of OMAS after probable COVID-19 infection with a complicated disease course over 3 months, including encephalopathy, seizures, and cognitive and psychiatric symptoms. After treatment with cortisone and IVIG, the patient made a full recovery. On the other hand, two patients showed signs of OMAS with isolated tremor–ataxia or opsoclonus and tremor. Both of these patients exhibited symptoms concurrent with COVID-19 infection or some days after and they quickly recovered without therapy. One patient had positive anti-GM2 and anti-sulfatide IgM antibodies. The patients had a work-up for paraneoplastic diseases and tumor screening without remarkable results.

In all patients, MRI and CT scans of the brain showed no lesions, and CSF revealed slightly elevated cell counts (ranging from 0 to 120 WBCs/mm^3^) and the results of COVID polymerase chain reaction (PCR) of the CSF were negative when performed.

All patients exhibited mild-to-severe COVID-19 symptoms, with three patients having severe and four having mild symptoms; five patients had an asymptomatic COVID-19 disease course.

**Fig. 1:**
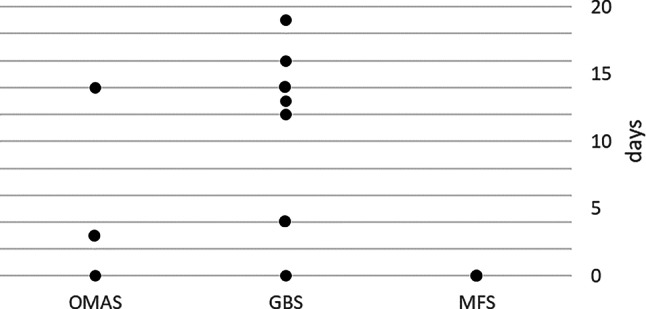
Time interval between COVID-19 infection and the onset of neurological symptoms for each subgroup. *GBS* Guillain–Barre syndrome, *MFS* Miller–Fisher syndrome, *OMAS* opsoclonus–myoclonus–ataxia syndrome

**Fig. 2:**
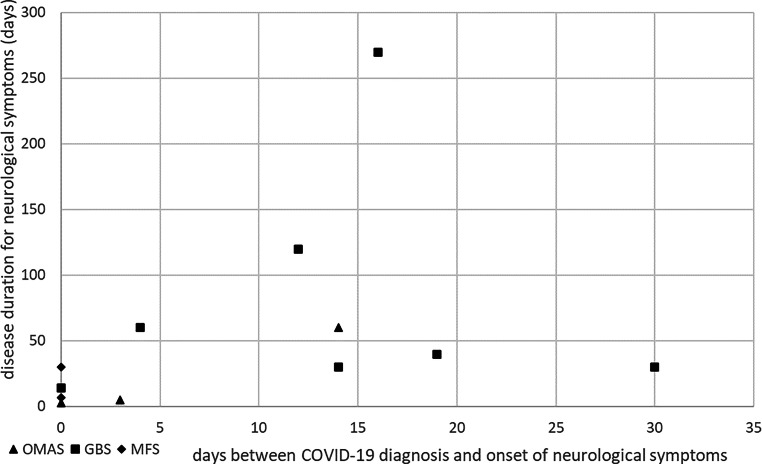
Scatterplot of time between COVID-19 symptom onset and neurological symptom onset in days (*x‑axis*) and disease duration in days (*y‑axis*).* GBS* Guillain–Barre syndrome, *MFS* Miller–Fisher syndrome, *OMAS* opsoclonus–myoclonus–ataxia syndrome

Figure 1 shows the time between COVID-19 infection and neurological symptom onset for each subgroup. Both patients with MFS had concurrent symptom onset and COVID-19 diagnosis. Figure 2 shows a scatterplot with time (in days) between infection diagnosis and the start of the neurological symptoms on the x‑axis and neurological disease duration in days on the y‑axis, suggesting a trend toward a shorter disease course until complete remission in patients with earlier onset of neurological symptoms.

## Discussion and conclusion

Here, we present patients with COVID-19-associated GBS, MFS, and OMAS treated during the COVID-19 pandemic in our hospital.

Clinical presentation of GBS and MFS patients correlated well with the existing literature. However, in our cohort, one of eight GBS patients and two of two MFS patients presented with a timely onset of GBS/MFS symptoms after the diagnosis of COVID-19, in contrast to the typical postinfectious onset described [2, 8]. In comparison, in the literature the time interval between COVID-19 infection and the onset of GBS varies in publications from 9 to 23 days [8, 9, 24]. However, late-onset GBS with a period of 33–45 days after COVID-19 infection has also been described, while in individual cases, GBS and COVID-19 were diagnosed concurrently [6].

Overall, two patients presented with concurrent COVID infection at symptom onset, and both quickly recovered within 2 weeks. All other patients had a prolonged disease course of up to 6 months. Two out of seven patients with GBS exhibited severe COVID-19 infection, requiring treatment in an intensive care unit, including mechanical ventilation. In the literature, mechanical ventilation was needed in 44% of GBS patients with COVID and in up to 30% of non-COVID-19 GBS patients [25]. In accordance with the current literature, all patients in our cohort showed a demyelinating phenotype of GBS [9].

Both patients with OMAS with symptom onset during SARS-Cov‑2 infection showed relatively mild symptoms and swift recovery without immunomodulatory treatment. One patient with a suspected SARS-CoV2 infection 2 weeks before symptom onset showed severe disease with encephalopathy over 3 months. Similar abortive symptoms in OMAS patients with concurrent onset have been suggested in individual cases [5].

Autoantibody positivity was observed in some patients: positive anti-GM2 and anti-sulfatide antibody in one patient with OMAS, anti-GT1A antibody in one patient with GBS, and anti-GT1A- and anti-GQ1b antibody in one patient with MFS. However, all other patients showed no autoantibodies. These results are supported by the literature for GBS and MFS patients [27].

While the results are in agreement with the existing literature, our main findings show two cases of abortive OMAS symptoms during acute COVID-19 infection, consistent with parainfectious disease. Although estimating the incidence of OMAS symptoms in COVID-19 patients is impossible in our case series, similar case numbers of OMAS, MFS, and GBS could indicate a higher incidence than previously suggested. Furthermore, in this case series, all subgroups presented with concurrent onset of SARS-CoV‑2 infection, contrary to inconsistent reports in the literature [16, 26]. Abbreviated neurological symptoms in the acute phase of COVID-19 infection can easily be missed if neurological evaluation is not readily available, as a delay in neurological assessment could lead to a missed diagnosis. Especially in patients with a severe COVID-19 disease course requiring mechanical ventilation and sedation, neurological symptoms can easily be missed.

### Limitations

The main limitation of the study lies in its single-center retrospective nature. In addition, not all COVID-19 patients were referred for an assessment by a neurological consultant, who would scan for specific neurological symptoms. Therefore, the number of unknown cases remains unclear.

## Conclusion

In summary, in this single-center retrospective cohort, a shorter disease duration was observed for parainfectious compared to postinfectious disease onset in patients with GBS, MFS, and OMAS. Furthermore, especially in the patients with OMAS, a faster recovery was observed in those with a parainfectious disease onset. This effect could be explained by different hypothesized pathomechanisms of para- and postinfectious onset. Further investigation in bigger cohorts would be necessary to determine the differences and possible pathomechanisms.

### Supplementary Information

Supplementary Table. The supplementary Table contains an overview over all included patients.
