# Supplementary material for: Retrospective analysis of COVID-19 patients with Guillain–Barre, Miller–Fisher, and opsoclonus–myoclonus–ataxia syndromes—a case series
Source: Wien Med Wochenschr. 2023 Jul 31;174(1-2):30–4. doi: 10.1007/s10354-023-01018-4 (PMC10810910; doi:10.1007/s10354-023-01018-4)
Supplement: Supplementary file 1 — Supplementary Table. The supplementary Table contains an overview over all included patients. [file 10354_2023_1018_MOESM1_ESM.docx]

Supplementary Table

| **Age** | **Sex** | **Syndrome** | **Symptoms** | **COVID status** | **COVID severity** | **Δt*** | **Duration of neurological symptoms** | **Treatment for neurological symptoms** | **CSF cell count per µl** | **CSF protein** | **autoantibodies** | **Relevant Medical history** |
| --- | --- | --- | --- | --- | --- | --- | --- | --- | --- | --- | --- | --- |
| 63 | m | OMAS | Opsoclonus-myoclonus-ataxia, cognitive and psychiatric symptoms, seizures | Probable COVID-19** | Mild | 14 days | worsening over 2 months, remission after 3 months | Steroid treatment, IVIG cycle | 6 C/µl | 70,2g/l | negative | COPD  MCI |
| 84 | m | OMAS | Tremor of lower extremities, ataxia | positive | Severe | 3 days | 5 days | none | 10 C/µl | 64,5g/l | negative | DM, TIA |
| 42 | m | OMAS | Opsoclonus, gaze-evoked nystagmus, tremor | positive | Mild | 0 days | 3 weeks | none | 2 C/µl | 73,4g/l | GM2-AB and Sulfatid-AB positiv | None |
| 53 | m | GBS  (AIDP) | Dysesthesia of face and legs, distal paresis of lower extremities | positive | Severe | 14 days | Residual symptoms after 4 weeks, lost to follow up | Steroid treatment | 0 C/µl | 70,3g/l | negative | None |
| 65 | w | GBS  (AIDP) | Paresis of lower extremities, dysesthesia | positive | Severe | 4 days | Residual symptoms after 2 months,  lost to follow up | IVIG cycle | 6 C/µl | 65,9g/l | n.p. | None |
| 48 | w | GBS  (AIDP) | Distal paraparesis | positive | Asymptomatic | 12 days | Remission within 6 months | IVIG cycle | 2 C/µl | 70,1g/l | GT1A-AB positive | None |
| 49 | m | GBS  (AIDP) | Facial palsy, dysesthesia, and hypesthesia, areflexia of lower extremities | positive | Asymptomatic | 0 days | Remission after 2 weeks | IVIG cycle | 3 C/µl | 69,0g/l | negative | None |
| 80 | w | GBS  (AIDP) | Tetra paresis, dysphagia, dysarthria, facial nerve palsy | positive | Mild | 19 days | Discharged to a rehab facility after 4 weeks | IVIG cycle | 67 C/µl | 69,9g/l | negative | DM |
| 60 | m | GBS  (AIDP) | Paraparesis | positive | Asymptomatic | 13 days | Remission after 30 days | IVIG cycle | 3 C/µl | 71,1g/l | negative | None |
| 40 | W | GBS  (AIDP) | Paraparesis, distal hypoestheisa | positive | Mild | 16 days | ongoing after 9 months, mild residual paräesthesia | IVIG cycle | 14 C/µl | 75,0g/l | negative | None |
| 34 | m | MFS | Ptosis, dysesthesia, areflexia | positive | Asymptomatic | 0 days | 1 week | IVIG cycle | 2 C/µl | 65,6g/l | negative | None |
| 49 | m | MFS | Complex oculomotor paresis | positive | Asymptomatic | 0 days | 1 month | none | 3 C/µl | 82,8g/l | GT1A-AB and GQ1b-AB positive | None |

n.p.: not performed.

MCI: myocardial infarction, COPD: chronic obstructive pulmonary disease, DM: Diabetes mellitus, TIA: Transient ischemic attack

AIDP: Acute inflammatory demyelinating polyneuropathy

*Δt: the interval between COVID-19 diagnosis and the onset of neurological symptoms

** According to WHO criteria

CSF Total protein: normal range 64-83g/l
